# C3 Drives Inflammatory Skin Carcinogenesis Independently of C5

**DOI:** 10.1016/j.jid.2020.06.025

**Published:** 2021-02

**Authors:** William D. Jackson, Alessandro Gulino, Liliane Fossati-Jimack, Rocio Castro Seoane, Kunyuan Tian, Katie Best, Jörg Köhl, Beatrice Belmonte, Jessica Strid, Marina Botto

**Affiliations:** 1 Centre for Inflammatory Disease, Department of Immunology and Inflammation, Imperial College London, United Kingdom; 2 Tumor Immunology Unit, Department of Health Sciences, University of Palermo School of Medicine, Palermo, Italy; 3 Department of Dermatology, Royal Victoria Infirmary, Newcastle Hospitals NHS Foundation Trust, Newcastle upon Tyne, United Kingdom; 4 Institute for Systemic Inflammation Research, University of Lübeck, Lübeck, Germany; 5 Division of Immunobiology, Cincinnati Children’s Hospital and College of Medicine, University of Cincinnati, Cincinnati, Ohio, USA

**Keywords:** CR, complement receptor, cSCC, cutaneous squamous cell carcinoma, DMBA, 7,12-dimethylbenz[a]anthracene, EC, epithelial cell, HPV16, human papillomavirus type 16, MAC, membrane attack complex, TPA, 12-O-tetradecanoylphorbol-13-acetate, WT, wild type

## Abstract

Nonmelanoma skin cancer such as cutaneous squamous cell carcinoma (cSCC) is the most common form of cancer and can occur as a consequence of DNA damage to the epithelium by UVR or chemical carcinogens. There is growing evidence that the complement system is involved in cancer immune surveillance; however, its role in cSCC remains unclear. Here, we show that complement genes are expressed in tissue from patients with cSCC, and C3 activation fragments are present in cSCC biopsies, indicating complement activation. Using a range of complement-deficient mice in a two-stage mouse model of chemically-induced cSCC, where a subclinical dose of 7,12-dimethylbenz[a]anthracene causes oncogenic mutations in epithelial cells and 12-O-tetradecanoylphorbol-13-acetate promotes the outgrowth of these cells, we found that C3-deficient mice displayed a significantly reduced tumor burden, whereas an opposite phenotype was observed in mice lacking C5aR1, C5aR2, and C3a receptor. In addition, in mice unable to form the membrane attack complex, the tumor progression was unaltered. C3 deficiency did not affect the cancer response to 7,12-dimethylbenz[a]anthracene treatment alone but reduced the epidermal hyperplasia during 12-O-tetradecanoylphorbol-13-acetate–induced inflammation. Collectively, these data indicate that C3 drives tumorigenesis during chronic skin inflammation, independently of the downstream generation of C5a or membrane attack complex.

## Introduction

Epithelial cells (ECs) line the surface of the skin and are therefore exposed to a variety of potential carcinogens, including chemicals and UV irradiation. Skin cancer is the most common of all cancers, with nonmelanoma skin cancers being by far the most frequent. UV irradiation is a well-known DNA-damaging agent of the skin, but chemical carcinogens are also prevalent in the environment, including polyaromatic hydrocarbons such as 7,12-dimethylbenz[a]anthracene (DMBA). The contribution of these carcinogens to the prevalence of nonmelanoma skin cancers is unclear. However, recent studies have shown that tobacco smokers are exposed to high levels of polyaromatic benz[a]anthracene and that their likelihood of developing cutaneous squamous cell carcinoma (cSCC) is increased by more than 50% (Leonardi-Bee et al., 2012).

The complement system is an important arm of innate immunity but also plays key roles in the activation and regulation of adaptive immunity (Merle et al., 2015a, Merle et al., 2015b). Complement can be activated through three pathways: the classical, the alternative, or the lectin pathway, with these pathways converging at the level of C3. Activation of C3 and subsequently C5 leads to the generation of C3a and C5a, both potent leukocyte chemoattractants and inflammatory mediators (Merle et al., 2015a, Merle et al., 2015b). Furthermore, C5 activation leads to the assembly of membrane attack complexes (MACs) that can lead to cell lysis (Fishelson and Kirschfink, 2019) or cell activation (Bohana-kashtan et al., 2004; Halperin et al., 1993; Morgan, 2016; Wagner et al., 1994). The role of the complement system in infection and autoimmunity is well-documented. More recently, its role in cellular metabolism (Kolev et al., 2015) and survival (Liszewski et al., 2013), tissue homeostasis, and immune surveillance is becoming recognized (Reis et al., 2019).

The skin has resident immune cells, which sense epithelial dysregulation and respond by activating a stress response to restore homeostasis (Crawford et al., 2018; Dalessandri and Strid, 2014). Development of skin cancer is underpinned by the accumulation of somatic mutations in ECs, but the fate of these mutated cells is strongly influenced by the surrounding microenvironment. Complement activation has traditionally been considered part of the cancer immune-surveillance response, and cancer cells have developed strategies to avoid complement-mediated destruction (Fishelson and Kirschfink, 2019; Macor et al., 2015). However, it is now widely accepted that many neoplastic conditions are driven by chronic and often subclinical inflammation, which can be accompanied by complement activation (Coussens et al., 2013; Diakos et al., 2014; Mantovani et al., 2008; Marx, 2004; Riihilä et al., 2017, 2015, 2014; Schonthaler et al., 2013). Once a malignant cell has escaped the early phase of immune surveillance, inflammation can exert prominent procarcinogenic effects. Therefore, in certain tumor microenvironments, the complement system may play a role in the promotion phase of carcinogenesis. In particular, the potent proinflammatory mediator C5a has been shown to contribute to tumor growth in models of cervical and lung cancer (Corrales et al., 2012; Markiewski et al., 2008; Nunez-Cruz et al., 2012), and inhibition of the receptor C5aR1, in combination with cytotoxic chemotherapy, can exert immunosuppressive effects in a papilloma virus‒induced mouse model of squamous carcinogenesis (Medler et al., 2018). However, conflicting results have been reported in other experimental models, indicating that the role of complement in tumor progression is multifaceted (Gunn et al., 2012).

In the context of the skin, C3 has been shown to be expressed in both human and mouse epidermis during inflammation (Giacomassi et al., 2017; Schonthaler et al., 2013). Equally, the complement regulator factors H and I are upregulated in human cSCC, supporting the notion of complement activation during skin carcinogenesis (Riihilä et al., 2015, 2014). In this study, we explored whether the complement system, which is produced and activated locally in the skin during tissue dysregulation, is involved in the protective skin immune-surveillance response against epithelial carcinogenesis. Using well-established models of chemically induced cSCCs, we show that C3 enhanced tumor susceptibility and tumor load in an inflammation-driven model but did not alter carcinogenesis in a model driven by mutational load. During tissue inflammation, we found that C3 acted as a cancer-promoting GF, independently of C3aR1, C5, and MAC. Thus, C3 activation, most likely through iC3b/C3b, contributes to the tissue inflammation, which drives the outgrowth of mutated ECs in the skin.

## Results

### C3 is expressed and activated in human skin tumors

**Figure 1:**
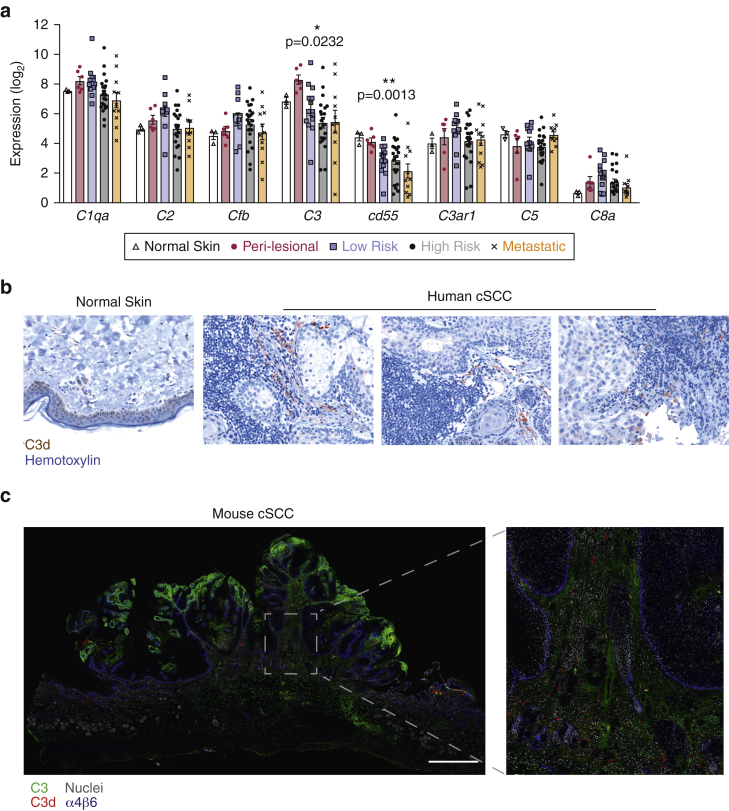
**C3 is activated in human and mouse skin tumors and correlates with disease severity.** (**a**) Expression of complement genes in (i) normal skin (n = 3), (ii) perilesional tissue (n = 6 donors), (iii) cSCC from patients with low-risk tumors (n = 12), (iv) high-risk tumors (n = 24), and (v) metastatic cSCC (n = 11). The transcriptomic data were obtained using NanoString and are presented as log_2_ values of RNA counts. Each symbol represents an individual donor. Statistical analysis was by one-way ANOVA and linear trend of expression between risk groups. (**b**) Representative micrographs showing C3d staining (brown) in human cSCC (n = 5) and normal skin (n = 2), counterstained with hematoxylin (blue). Original magnification is ×200. (**c**) Representative micrograph of C3 (green), C3d (red), integrin α4β6 (blue), and nuclei (gray) in a mouse cSCC from the DMBA-TPA model. Bar = 1 mm. cSCC, cutaneous squamous cell carcinoma; DMBA, 7,12-dimethylbenz[*a*]anthracene; TPA, 12-O-tetradecanoylphorbol-13-acetate.

To investigate the role of complement in cSCC, we performed gene expression analysis of complement genes in a cohort of 56 patients with cSCC. Samples were independently divided into risk groups according to tumor severity. Normal skin and perilesional tissue were also included in the analysis. We observed a linear negative correlation between mRNA expression of *C3* and *Cd55,* a key regulator of C3 convertase, in normal or perilesional skin samples and samples with more advanced cSCC (Figure 1a), indicating that in these tumors, the analysis of C3 synthesis might not provide an accurate assessment of its activation. Of note, other complement components did not display any significant association, indicating no specific contribution from the classical (*C1qa*), lectin (*C2*), alternative (*Cfb*), or the terminal pathways (*C5* and *C8a*). Furthermore, there was no association between the degree of disease risk and the level of *C3aR1* mRNA (Figure 1a), making the involvement of this pathway in tumor progression unlikely.

Given the transcriptional association between *C3* and *Cd55* with human cSCC severity, we next assessed whether C3 is deposited and activated within human cSCC tissue. We detected hardly any C3d in normal skin (Figure 1b) but extensive C3d deposition at the edge of the cSCC tumor mass, mainly in close proximity to tumor-infiltrating mononuclear cells, most likely myeloid cells (Figure 1b). Overall, these in-situ analyses confirmed C3 expression and activation within the tumor microenvironment of cSCC, suggesting its contribution to cancer development.

### C3 supports tumor growth in an inflammation-driven skin carcinogenesis model

**Figure 2:**
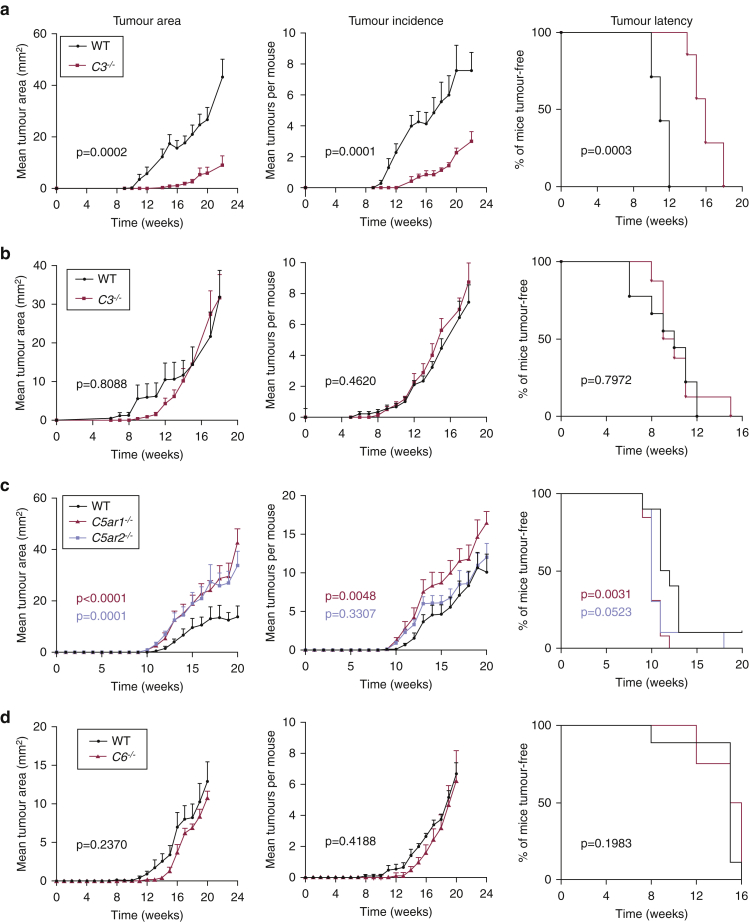
**The tumor-promoting effect of C3 depends on local chronic inflammation and does not require C5 activation.** (**a**) Tumor susceptibility to DMBA-TPA carcinogenesis in *C3*^*−/−*^BALB/c (red lines, n = 7) and WT mice (black lines, n = 7). (**b**) Tumor susceptibility to DMBA-only carcinogenesis in *C3*^*−/−*^BALB/c (red lines, n = 8) and WT mice (black lines, n = 9). (**c**) Tumor susceptibility to DMBA-TPA carcinogenesis in *C5ar1*^*−/–*^ (red lines, n = 10) and *C5ar2*^*−/–*^ (blue lines, n = 10) mice versus WT mice (black lines, n = 9). (**d**) Tumor susceptibility to DMBA-TPA carcinogenesis in *C6*^*−/−*^ mice (red lines, n = 8) versus WT mice (black lines, n = 10). (**a–d**) Data presented as tumor latency (frequency of tumor-free mice), tumor incidence (mean number of tumors), and tumor area (mean tumor size). Statistical analysis of tumor latency curves by Mantel‒Cox test and tumor load by linear regression; *P*-values are indicated. Data are representative of two independent experiments for each strain. DMBA, 7,12-dimethylbenz[*a*]anthracene; TPA, 12-O-tetradecanoylphorbol-13-acetate; WT, wild type.

The presence of C3 activation products in human cSCC tissues led us to evaluate the contribution of this molecule to tumor growth in the two-stage DMBA‒12-O-tetradecanoylphorbol-13-acetate (TPA) mouse model of skin carcinogenesis, where *EC* mutations are caused by exposure to a subclinical dose of carcinogen (DMBA) and outgrowth of tumors occurs after a prolonged inflammation induced by topical TPA treatment. Consistent with our human data, staining of DMBA-TPA‒induced tumors revealed extensive C3 expression both in the peritumoral infiltrate and in the epithelial tumor mass as well as in the underlying dermis (Figure 1c). In contrast, C3d deposition was mostly limited to the peritumoral compartment (Figure 1c). Guided by these observations, we investigated the tumor susceptibility of C3-deficient (*C3*^*−/−*^) mice and found that these mice were markedly protected from tumor development (Figure 2a). After 22 weeks of tumor promotion with TPA, the *C3*^*−/−*^ mice developed significantly fewer and smaller tumors than the corresponding wild-type (WT) mice (Figure 2a). *C3*^*−/−*^mice also had a slower tumor onset time; by week 12 of TPA, 100% of the WT mice had developed tumors, whereas *C3*^*−/−*^mice were still tumor free (Figure 2a). We next tested whether this tumor-promoting effect of C3 could be explained by differences in the response to the carcinogen DMBA. However, when we examined tumor susceptibility in a model of carcinogenesis driven exclusively by repeated exposure to DMBA, there was no effect of C3 (Figure 2b). Tumor area, tumor incidence, and tumor onset time were similar, suggesting that the protumorigenic effect of C3 occurred during the chronic inflammatory phase.

**Supplementary Figure S1:**
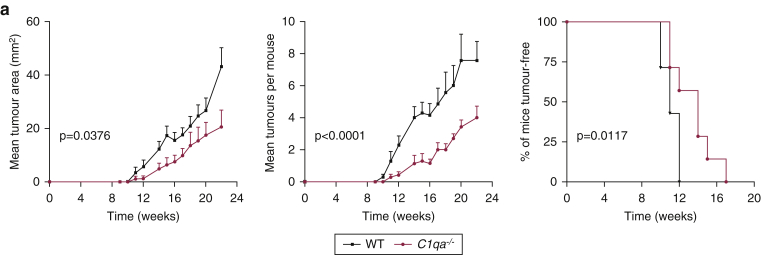
**Effect of C1q on DMBA-TPA carcinogenesis.** (a) Tumor susceptibility to DMBA-TPA carcinogenesis in *C1q*^*−/−*^ (red lines, n = 7) mice versus WT (black lines, n = 7). Data are presented as tumor latency (frequency of tumor-free mice over time), tumor incidence (mean number of tumors per mouse), and tumor area (mean tumor size per mouse). Statistical analysis of tumor latency curves was done by Mantel‒Cox test and tumor load by linear regression; *P*-values are indicated. The representative of a single experiment is shown. DMBA, 7,12-dimethylbenz[*a*]anthracene; TPA, 12-O-tetradecanoylphorbol-13-acetate; WT, wild type.

Because C3 activation leads to the generation of C5a, which is a potent proinflammatory mediator, and to the formation of the MAC, we next explored the role of these pathways using mice lacking the receptors for C5a (C5aR1 or C5aR2) and mice lacking C6. Unexpectedly, the tumor area was increased in *C5ar1*^*−/−*^ (*P* < 0.0001) and *C5ar2*^*−/−*^ (*P* = 0.0001) mice compared with WT controls (Figure 2c). *C5ar1*^*−/−*^ mice also showed a mild increase in tumor number (*P* = 0.0048) and tumor onset time (*P* = 0.0031), whereas these parameters were unaltered in *C5ar2*^*−/−*^ mice (Figure 2c). Contrarily, C6-deficient (*C6*^*−/−*^) mice, which are unable to form the MAC, exhibited no abnormalities in either tumor incidence, size, or latency compared with WT controls (Figure 2d). Interestingly, C1q-deficient mice, which are unable to activate the classical pathway, also showed a reduced tumor growth, but the degree of protection was less pronounced than in the *C3*^*−/−*^ mice, indicating that the activation of C3 was only partially mediated through the classical pathway (Supplementary Figure S1a). These data collectively show that C3 promotes inflammation-driven outgrowth of skin tumors independently of the downstream generation of C5a or MAC.

### C3 promotes epidermal hyperplasia during skin inflammation

**Figure 3:**
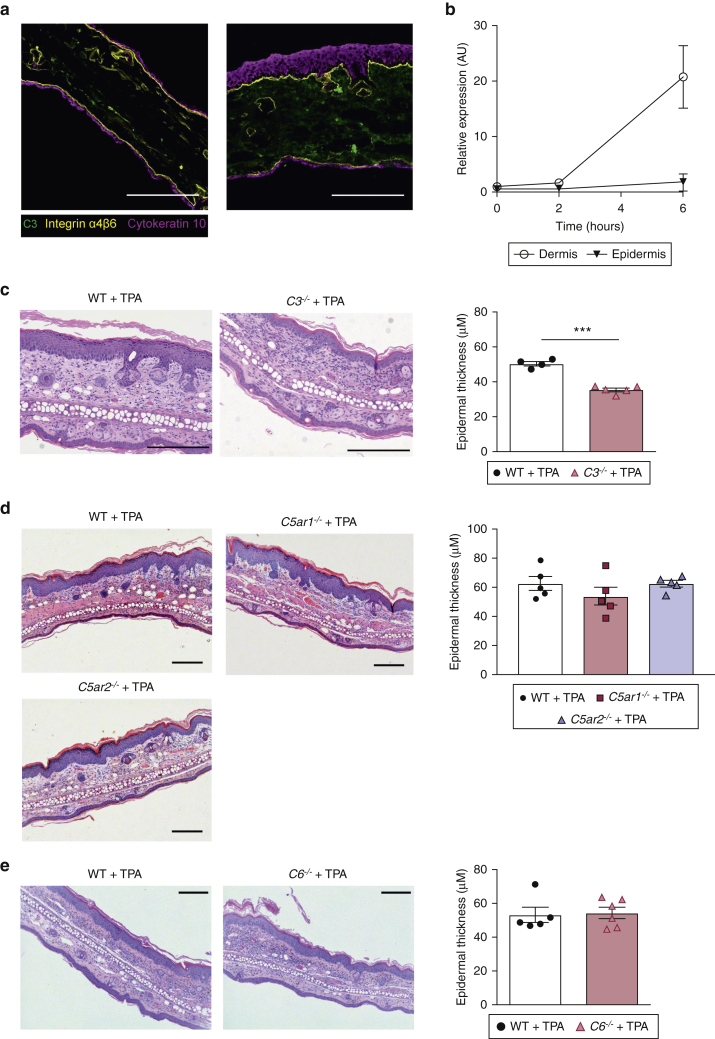
**C3 deposits in inflamed skin and promotes epidermal hyperplasia independently of C5a.** (**a**) Representative staining of C3 (green), cytokeratin 10 (magenta), and integrin α4β6 (yellow) in naïve and 4×TPA‒treated skin from WT mice. Bar = 200 μm. (**b**) qRT-PCR of *C3* transcript in dermis and epidermis of WT mice (n = 3), either untreated or after 1× topical TPA. (**c–e**) H&E stained ear skin after 4×TPA in indicated complement-deficient mice with quantification of epidermal thickness. Bar = 200 μm. (**c**) WT (n = 4) and *C3*^*−/−*^ (n = 5) mice. A representative of two independent experiments is shown. Statistical analysis was done by two-tailed Student’s *t*-test. (**d**) WT (n = 5), *C5ar1*^*−/−*^ (n = 6), and *C5ar2*^*−/−*^ (n = 6) mice. A representative of two independent experiments is shown. Statistical analysis was by one-way ANOVA with Tukey’s multiple comparison. (**e**) WT (n = 5) and *C6*^*−/−*^ (n = 6) mice. A representative of a single experiment is shown. Statistical analysis by one-way ANOVA with Tukey’s multiple comparison, ^∗∗∗^indicates a *P*-value <0.001. AU, arbitrary units; qRT-PCR, quantitative real-time reverse transcriptase–PCR; TPA, 12-O-tetradecanoylphorbol-13-acetate; WT, wild type.

To further understand how C3 might be promoting tumor development in an inflammatory microenvironment, we investigated the role of C3 during TPA-driven skin inflammation without the application of carcinogen. We detected enhanced C3 deposition in the dermis when TPA was applied repeatedly (four times over 2 weeks) on the dorsal ear skin (Figure 3a). An increase of *C3* transcript, especially in the dermis, was already detectable at 6 hours after a single TPA treatment, indicating that C3 protein was being synthesized locally (Figure 3b). Histological examination of the ear skin revealed that the epidermal hyperplasia triggered by the TPA application was significantly reduced in *C3*^*−/−*^ mice **(**Figure 3c). To confirm that this proliferative effect on skin ECs was mediated by C3 only and not the result of downstream complement activation effects, we exposed *C5ar1*^*−/−*^, *C5ar2*^*−/−*^ (Figure 3d), and *C6*^*−/−*^ (Figure 3e) mice to the same TPA treatment and found no difference in epidermal hyperplasia compared with controls. Taken together, these results indicate that only C3 plays a key role in EC growth and the development of epidermal hyperplasia during TPA-induced skin inflammation.

### The effect of C3 on epidermal hyperplasia is neutrophil independent

**Figure 4:**
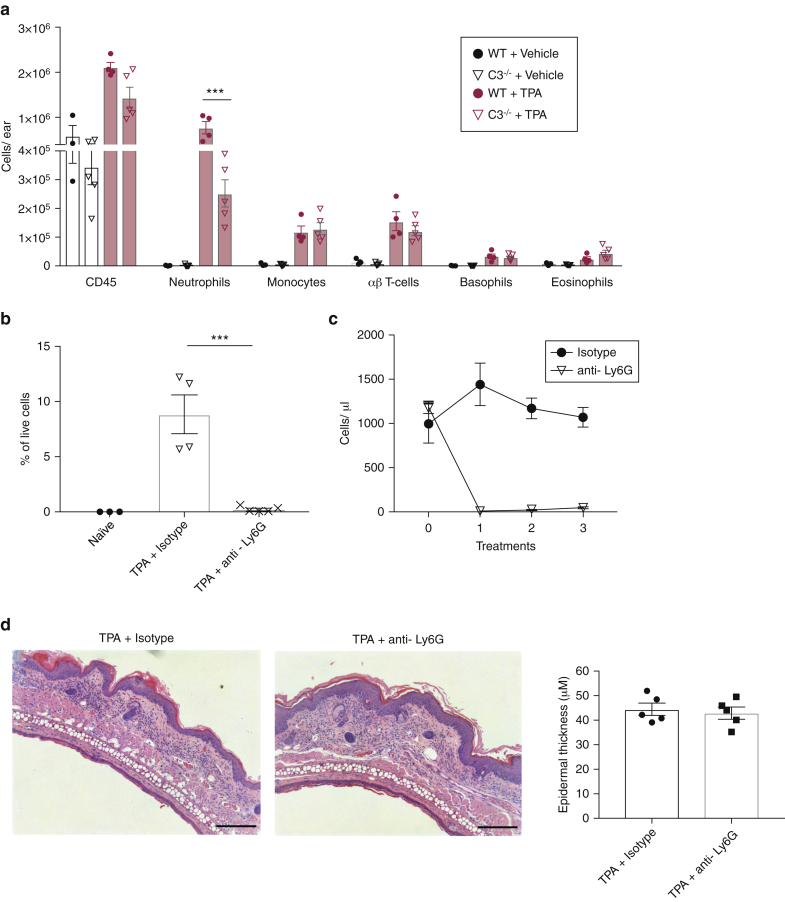
**C3-mediated neutrophil recruitment does not contribute to inflammation-induced epidermal hyperplasia.** (**a**) Dorsal ear skin from WT and *C3*^*−/−*^ mice was treated topically with vehicle (WT: n = 3, *C3*^*−/−*^*:* n = 4) or 4×TPA (WT: n = 4, *C3*^*−/−*^*:* n = 5), and leukocyte infiltrate was analyzed by flow cytometry. Populations were quantified as cell counts per ear. (**b–d**) WT mice were treated topically with 4×TPA. Simultaneous to TPA treatment, mice received an i.p. injection of anti-Ly6G antibody (IA8) or isotype antibody (n = 5). (**b**) Percentage of neutrophils (gated CD45^+^, CD11b^high^, Ly6c^int^) in the ear skin. Statistical analysis was by one-way ANOVA with Tukey’s multiple comparison. (**c**) Neutrophils per μl blood at 24 hours after each antibody treatment. (**d**) Quantification of epidermal thickness and representative histological images. Bar = 200 μm. Statistical analysis by (**a, b**) one-way ANOVA with Tukey’s multiple comparison and (**d**) two-tailed Student’s *t*-test, ^∗∗∗^indicates a *P*-value <0.001. i.p., intraperitoneal; TPA, 12-O-tetradecanoylphorbol-13-acetate; WT, wild type.

**Supplementary Figure S2:**
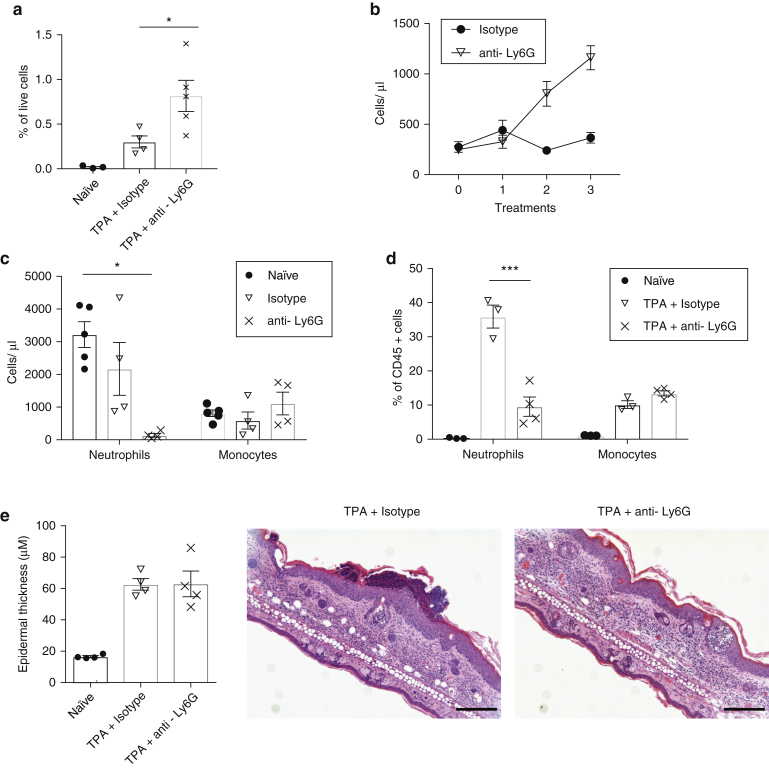
**Neutrophils do not contribute to TPA-induced epidermal hyperplasia.** (**a, b**) WT mice were treated topically with 4×TPA. Simultaneous to TPA treatments, mice received an i.p. injection with chimeric anti-Ly6G antibody (IA8) or an isotype antibody; n = 5 per group. (**a**) Percentage of monocytes (gated as CD45^+^, CD11b^+^, Ly6c^high^ from live cells) in dorsal ear skin. (**b**) Monocytes per μl blood at 24 hours after each treatment. (**c–e**) The same experimental design was performed using a rat IgG1 anti-Ly6G (IA8) or isotype antibody; n = 3–5 per group. (**c**) Neutrophils (gated CD45^+^, CD11b^+^, CD115^–^) and monocytes (gated CD45^+^, CD11b^+^, CD115^+^, Ly6G^−^) per μl blood after two treatments. (**d**) Percentage of neutrophils (gated CD45^+^, CD11b^high^, Ly6c^int^) and monocytes (gated CD45^+^, CD11b^int^, Ly6c^high^) of total leukocytes in ear skin of animals treated with 4× topical TPA receiving rat IgG1 isotype or IA8. Naïve mice were used as controls. (**e**) H&E stained ear skin after 4×TPA treatment and quantification of epidermal thickness. Bar = 200 μm. Statistical analysis in all panels is by one-way ANOVA with Tukey’s multiple comparison. i.p., intraperitoneal; TPA, 12-O-tetradecanoylphorbol-13-acetate; WT, wild type.

To understand whether C3 promotes epidermal hyperplasia by affecting the inflammatory immune infiltrate, we performed FACS analysis of the TPA-treated ear skin from *C3*^*−/−*^ mice and WT controls. Topical applications of TPA induced a robust leukocyte infiltration into the skin, with neutrophils, monocytes, and αβ T cells being the most abundant cell populations (Figure 4a). The overall infiltration of CD45^+^ leukocytes was not altered in the absence of C3; however, we observed a significantly decreased number of neutrophils in *C3*^*−/−*^ skin compared with WT skin (mean 7.74 × 10^5^ vs. 2.52 × 10^5^, *P* = 0.0007) (Figure 4a). The recruitment of other immune cell populations was not significantly altered (Figure 4a). Given the effect of C3 activation on neutrophil recruitment, we tested whether these cells were responsible for driving the epidermal hyperplasia by depleting neutrophils before the TPA application using a mouse anti-Ly6G antibody (Daley et al., 2008). Injection of this antibody achieved a complete depletion of neutrophils in the skin after four TPA treatments (Figure 4b) and in the blood (Figure 4c) at 24 hours after the first administration, which persisted throughout the experiment (Figure 4c). Histological analysis of the TPA-treated skin showed that neutrophil depletion had no effect on epidermal thickness (Figure 4d), demonstrating that EC proliferation was not influenced by the presence of neutrophils in the dermis. Of note, injection of the anti-Ly6G antibody caused a degree of monocytosis in the skin (Supplementary Figure S2a) and peripheral blood (Supplementary Figure S2b), a phenomenon that has previously been reported (Patel et al., 2019). To address this confounding effect, we performed the neutrophil depletion again using a rat IgG2a isotype of the anti-Ly6G antibody. This depleted the blood neutrophils (Supplementary Figure S2c) and skin neutrophils (Supplementary Figure S2d), albeit less efficiently than the chimeric mouse IgG2a antibody, but did not induce monocytosis (Supplementary Figure S2d). Once again, epidermal thickening after TPA was unaffected by the removal of neutrophils (Supplementary Figure S2e).

### The effect of C3 on tumor growth and epidermal hyperplasia is independent of C3aR1

**Figure 5:**
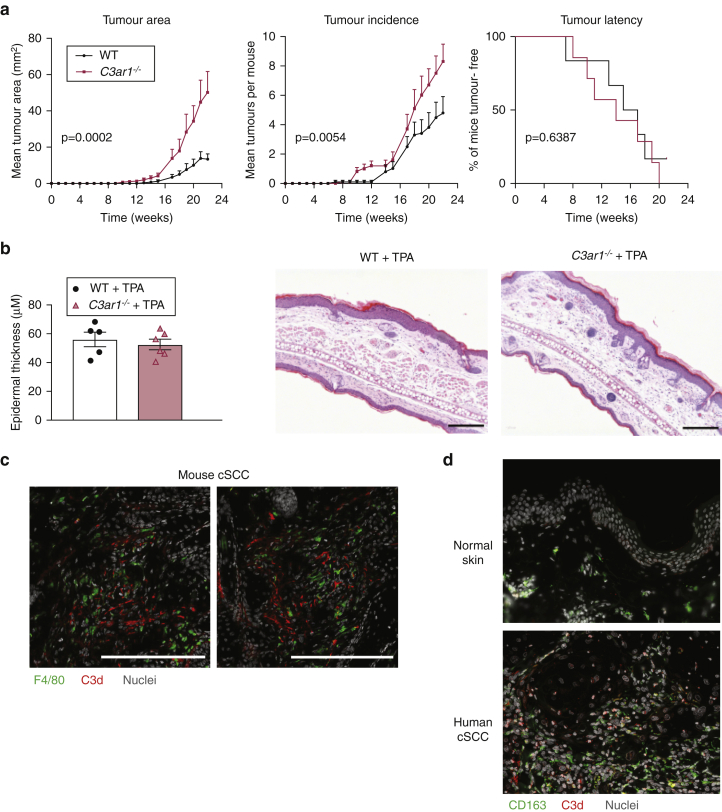
**Tumor progression and epidermal hyperplasia are independent of C3aR1.** (**a**) Tumor susceptibility to DMBA-TPA carcinogenesis in *C3ar1*^*–/–*^ (red lines, n = 10) and WT mice (black lines, n = 10). Data are presented as tumor latency, tumor incidence, and tumor area. Statistical analysis of tumor latency was by Mantel‒Cox test and tumor load by linear regression; *P*-values are indicated. Data representative of two independent experiments are shown. (**b**) H&E stained ear skin after treatment with 4×TPA in WT (n = 5) and *C3ar1*^*−/−*^ (n = 6) mice with quantification of epidermal thickness. Bar = 200 μm. (**c**) Representative immunofluorescence of C3d (red) and F4/80 (green) in DMBA-TPA‒induced mouse cSCC. Nuclei are counterstained with Hoescht 33342 (gray). Bar = 200 μm. (**d**) Representative immunofluorescent micrographs of C3d (red) and CD163 (green) staining in human cSCC (n = 5) and normal skin (n = 2). Nuclei are counterstained with DAPI (gray). Original magnification is ×200. cSCC, cutaneous squamous cell carcinoma; DMBA, 7,12-dimethylbenz[*a*]anthracene; TPA, 12-O-tetradecanoylphorbol-13-acetate; WT, wild type.

Given that C3 promotes both cutaneous carcinogenesis (Figure 2) and epidermal hyperplasia during skin inflammation (Figure 3) independently of downstream C5 activation, we hypothesized that these effects could be mediated by C3a signaling through the C3aR1. Therefore, we utilized mice deficient in this receptor and analyzed their susceptibility to tumor development and epidermal hyperplasia. Surprisingly, when *C3ar1*^*−/−*^ mice underwent DMBA-TPA carcinogenesis, we observed an increase in mean tumor area and tumor incidence, although there was no significant difference in the time to tumor onset (Figure 5a). Furthermore, when we induced skin inflammation with TPA only, we observed no significant difference in epidermal thickness between WT mice and those lacking C3aR1 (Figure 5b)*,* consistent with the modest phenotype observed in the carcinogenesis model. These findings indicate that the effect of C3 on tumor growth was unlikely to be mediated by C3aR1 and suggest that other pathways and/or mechanisms triggered by C3b/iC3b support skin epithelial growth. Given the strong expression of complement receptors (CR) such as CR3 and CR4 on mononuclear phagocytes, we performed double immunostaining for macrophages and C3d, a complement activation product that remains bound on the cell surface, to determine colocalization. In both mouse **(**Figure 5c) and human (Figure 5d) specimens, we found that C3d-positive cells were in very close proximity to macrophages (F4/80^+^ in mouse and CD163^+^ in human samples), indicating that complement activation may orchestrate the interactions between the phagocytic cells and the tumor cells with important implications for tumor progression.

## Discussion

The data presented in this study define a key role for C3, the central component of the complement system, in the onset and growth of cSCC. We found C3 activation products in both human and mouse tumors in close proximity to infiltrating macrophages. The absence of C3 strongly protected against skin cancer development in the inflammation-driven DMBA-TPA model of cSCC but not in the absence of the inflammatory stimulus. Intriguingly, the deficiency of C3aR1, C5aR1, or C5aR2 generated opposite results to the deficiency of C3, with a modest increase in tumor number and growth, whereas the MAC did not play a key role either way. Together these findings suggest that in an inflammatory microenvironment, C3 can promote tumor growth by fueling epithelial hyperplasia, most likely through pathways mediated by iC3b/C3b/C3d on infiltrating myeloid cells.

Deposits of complement components have been reported in various human tumors (Roumenina et al., 2019). Consistent with other reports (Bonavita et al., 2015; Riihilä et al., 2017), we found in both human and mouse skin tumors an abundance of C3 activation products, specifically C3d, presumably marking cells ready for disposal by mononuclear phagocytes. One of the key C3d receptors present on myeloid cells is CR3, consisting of CD11b and CD18. It has been demonstrated in multiple contexts that binding of complement-opsonized cells to CR3 is an immune-inhibitory signal, which is necessary to induce tolerance (Sohn et al., 2003). In the tumor microenvironment, iC3b released from apoptotic tumor cells can prevent the maturation of dendritic cells and promote cancer progression (Schmidt et al., 2006). In addition, CR3 ligation can negatively impact tumor immune surveillance through the direct inhibition of NK cell‒mediated cytotoxicity (Liu et al., 2017). Our data showing a strong protective effect of C3 deficiency in cSCC would be consistent with the idea that C3 activation fragments can act as tumor-promoting GFs by orchestrating the immune microenvironment and modulating the adaptive immune response. Studies to discriminate between the innate and adaptive effects remain challenging because CR3 is an integrin involved in a diverse range of leukocyte functions.

To understand how the complement cascade is activated in human cSCC, we analyzed the transcriptomic profile of patients with cSCC at different stages of progression. The data did not identify the upregulation of a specific complement activation pathway. Our mouse data showing that C1q deficiency provided less protection than C3 deficiency in the DMBA-TPA carcinogenesis model would support the concept that in cSCC, complement activation may occur, at least in part, independently of antibodies. The human transcriptomic data also revealed a prognostic negative effect of low expression of C3 and CD55 (a key regulator of C3 convertase, and thus, its downregulation would increase C3 activation). These two findings seem to be in contradiction to each other, but most likely, the lower *C3* mRNA level in more advanced tumors just reflects a reduced number of infiltrating immune cells because these are the main sources of C3.

C3a and C5a are inflammatory anaphylatoxins that act as chemokines to mediate myeloid cell recruitment as well as cause histamine release from mast cells, vasodilation, and increased vascular permeability (Klos et al., 2009). Both of these inflammatory pathways were protective against inflammation-driven skin carcinogenesis. This suggests that myeloid cell recruitment to the skin may be necessary to control tumor growth. C5a has two receptors, C5aR1 and the less-studied C5aR2. The canonical view is that C5aR2 is a decoy receptor for C5aR1, which lacks G-protein binding and is internalized upon ligation, thus contributing to the hypothesis that C5aR2 clears excess C5a to resolve inflammation (Gerard et al., 2005; Scola et al., 2009). However, in our skin tumor model, the lack of either receptor promoted tumor susceptibility. The fact that the net effect of C5aR1 and C5aR2 ligation had a similar effect may be explained by the discovery that C5aR2 can form heteromers with C5aR1 and modulate downstream signaling through the recruitment of β-arrestin (Croker et al., 2014). This heterodimerization may also offer an explanation for the disease models in which C5aR2 deficiency alone has been shown to be protective (Hao et al., 2013; Poppelaars et al., 2017; Rittirsch et al., 2008) or where the effects of C5aR1 and C5aR2 are synergistic (Kovtun et al., 2017; Martin et al., 2018). Interestingly, C5aR2 has also been shown to modulate signaling downstream of C3a through an unknown mechanism (Chen et al., 2007), offering another potential explanation for the similarity of our results using the C5aR2- and C3aR1-deficient mice.

Our data showing that C3, independent of C5 and C6, promotes the onset and growth of cSCC is in contrast to the findings in the human papillomavirus type 16 (HPV16)-driven squamous carcinoma model (de Visser et al., 2004). Coussens et al. (2013) have suggested that cSCC progression is independent of C3 and driven by C5a through C5aR1 (de Visser et al., 2004). There are a number of key differences that may explain these discrepancies. Coussens et al. (2013) utilize the K14-HPV16 transgenic model of epithelial carcinogenesis in which the expression of HPV16 promotes epidermal dysplasia and development of nonmelanoma skin tumors (Arbeit et al., 1994). Mast cells extensively infiltrate these tumors and are crucial for disease progression, partially through their expression of C5aR1 and activation by C5a (Medler et al., 2018). In contrast, we observed only a few mast cells in the skin during TPA-induced hyperplasia and an almost complete absence of mast cells in the DMBA-TPA–induced tumors (Hayes et al., 2020) and together with other studies, found no clear role for mast cells in cSCC development (Antsiferova et al., 2013; Hayes et al., 2020). Human epidemiological studies support a clear link between dermal exposure to polyaromatic hydrocarbons such as DMBA and skin cancer (Siddens et al., 2012). In contrast, the link between HPV16 and skin cancer is less clear (Ma et al., 2014). Our results are consistent with the findings reported by another group (Bonavita et al., 2015) and the data obtained by knocking down *C3* in an in vivo model of human cSCC xenografts (Riihilä et al., 2015). In the K14-HPV16 model, Medler et al. (2018) demonstrate that C5a is released independently of C3 to promote immunosuppression and that C5aR1 blockade can increase responsiveness to immunotherapy, although there was no effect of C5aR1 blockade alone. In the DMBA-TPA data presented here, C5aR1 deficiency was in fact detrimental to disease progression, clearly indicating a different disease mechanism in the two models.

In summary, our data show that C3 drives squamous carcinoma cell tumorigenesis during chronic skin inflammation, independently of the downstream generation of C5a or MAC. Given that this phenotype does not rely on C3aR1 and the abundance of macrophages in association with C3 activation products, this tumorigenic effect is most likely driven through a C3b/iC3b-CR3 axis on myeloid cells. Further work is required to elucidate this mechanistic link, which may represent a novel therapeutic strategy in cSCC.

## Materials and Methods

For extended Materials and Methods, refer to Supplementary Materials and Methods.

### Mice

The following complement-deficient strains, backcrossed to BALB/c, were used: *C3*^*−/−*^ (Wessels et al., 1995), *C1qa*^*−/−*^ (Botto et al., 1998), C5ar1^*−/−*^ (Höpken et al., 1996), *C5ar2*^*−/−*^ (Gerard et al., 2005), and *C3ar1*^*−/−*^ (Humbles et al., 2000). C6^*−/−*^ mice were on a C57BL/6 background (Bhole and Stahl, 2004). All procedures were approved by the Imperial College Animal Welfare and Ethical Review Body committee, the United Kingdom Home Office and conducted in accordance with the Animal Research: Reporting of In Vivo Experiments guidelines (Percie du Sert et al., 2020).

### Chemical-induced skin inflammation and carcinogenesis

Chemicals DMBA and TPA were purchased from Sigma-Aldrich (St. Louis, MI). Skin inflammation was induced by topical exposure of the dorsal sides of the ear skin to a single or repeated doses of 2.5 nmol of TPA. Chemical carcinogenesis protocols were conducted as previously described (Strid et al., 2008).

### Neutrophil depletion

To deplete blood neutrophils, anti-Ly6G antibody was injected intraperitoneally as previously described (Daley et al., 2008). Mice were injected with 200 μg of either rat IgG2a anti-Ly6G (Clone 1A8, Biolegend, San Diego, CA) or a chimeric anti-Ly6G composed of the 1A8-variable region with a mouse IgG2a fragment-crystallizable region (Absolute Antibody, Redcar, United Kingdom).

### Histology and immunofluorescence of mouse skin

Formalin-fixed, paraffin-embedded ear skin sections were stained with H&E, and images were acquired using a Leica DM4B microscope (Leica Microsystems, Wetzlar, Germany). Epidermal thickness was measured using ImageJ (Schindelin et al., 2012).

Tissue was harvested and snap frozen in optimal-cutting temperature. The 9 μM sections were stained with antibodies detailed in Supplementary Materials and Methods. Hoescht 33342 (NucBlue, ThermoFisher, Waltham, MA) was added to the final staining step, and sections were mounted in ProLong Glass (ThermoFisher).

### Immunohistochemical and immunofluorescent staining of human skin

Immunohistochemistry was carried out on formalin-fixed, paraffin-embedded human tissue sections. Tissue samples were incubated with a rabbit polyclonal anti-C3d antibody (1:100 pH9; Cell Marque, Rocklin, CA, Code 403A-76). The immunostaining was revealed by a polymer-detection method (Novolink Polymer Detection Systems, Leica Biosystems, product number RE7280-K) and counterstained with Harris hematoxylin (Novocastra, Newcastle upon Tyne, United Kingdom).

For immunofluorescence, sections were stained with the antibodies detailed in Supplementary Materials and Methods. Nuclei were counterstained with DAPI.

### Quantitative real-time reverse transcriptase–PCR and primer sequences

Ear skin was split into the dorsal and ventral side, and the dermis was separated from the epidermis using 0.5 M ammonium thiocyanate. RNA was extracted with an RNeasy Mini Kit (Qiagen, Hilden, Germany). Complementary DNA was synthesized from RNA using an iScript synthesis kit (Bio-Rad, Hercules, CA). qRT-PCR products were detected with SYBR Green (ThermoFisher) measured continuously with a ViiA 7 Real-Time PCR system (Applied Biosystems, Foster City, CA). Target gene expression was normalized to the mean of two housekeeping genes encoding GAPD and 18S ribosomal RNA. Primer sequences are listed in Supplementary Materials and Methods.

### Nanostring analysis

For gene expression in squamous cell carcinoma tumors, formalin-fixed, paraffin-embedded skin samples were obtained from 56 patients at the University of Dundee (United Kingdom), Tayside National Health Service Trust, and Greater Glasgow and Clyde National Health Service Trust. The study was conducted according to the Declaration of Helsinki Principles, and all patients donating samples to this study provided written informed consent in accordance with ethical approval from the East of Scotland Research Ethics Service REC 1. Total RNA was extracted using a Qiagen RNeasy extraction kit (Qiagen) and was directly hybridized using the NanoString PanCancer and PanCancer Immune expression panel and analyzed on an nCounter (NanoString, Seattle, WA).

### Tissue digestion and flow cytometry

Single-cell suspensions were obtained by tissue digestion using 25 μg/ml Liberase (Roche, Basel, Switzerland) and 250 μg/ml DNAseI (Roche). Cells were then incubated with 2.4G2 mAb to block nonspecific fragment-crystallizable receptor binding and incubated with a live and/or dead discrimination dye (LIVE/DEAD Fixable Dead Cell Stain, ThermoFisher), followed by the fluorochrome-conjugated antibodies listed in Supplementary Table S1. For blood samples, red blood cells were removed using BD FACS Lysing Solution (BD Biosciences, Franklin Lakes, NJ) before staining. Samples were analyzed on a Fortessa ×20 flow cytometer (BD Biosciences).

### Statistical analysis

Experimental groups were compared using two-tailed Student’s *t*-test for unpaired data. For experiments with more than two groups, unpaired data were analyzed using one-way ANOVA with Tukey’s multiple comparison. Tumor burden between groups was assessed by linear regression, whereas tumor onset was analyzed using Mantel‒Cox test. Asterisks denote significance as follows: ∗*P* < 0.05; ∗∗*P* < 0.01; ∗∗∗*P* < 0.001; and ∗∗∗∗*P* < 0.0001. Statistical tests were performed using GraphPad Prism 7.0 for Mac (GraphPad Software, La Jolla, CA).

### Data availability statement

No datasets were generated or analyzed during this study.

## ORCIDs

William D. Jackson: http://orcid.org/0000-0002-2718-8117

Alessandro Gulino: http://orcid.org/0000-0002-2579-4519

Liliane Fossati-Jimack: http://orcid.org/0000-0003-3757-3999

Rocio Castro Seoane: http://orcid.org/0000-0002-2938-3872

Kunyuan Tian: http://orcid.org/0000-0001-5163-6381

Katie Best: http://orcid.org/0000-0002-1745-6392

Jörg Köhl: http://orcid.org/0000-0003-1121-3178

Beatrice Belmonte: http://orcid.org/0000-0001-9668-0925

Jessica Strid: http://orcid.org/0000-0003-3690-2201

Marina Botto: http://orcid.org/0000-0002-1458-3791

## Conflict of Interest

The authors state no conflict of interest.

## Figures and Tables

**Supplementary Table S1:** Fluorochrome-Conjugated Antibodies Used for Cell-Surface Staining

Antigen	Clone	Manufacturer
IgE	RME-1	Biolegend
CD117	2B8	Biolegend
CD11b	M1/70	eBioscience
Ly6G	1A8	Biolegend
Ly6C	HK1.4	Biolegend
CD45	30-F11	eBioscience
CD115	AFS98	Biolegend
Siglec F	E50-2440	BD Biosciences
TCR β	H57-957	Biolegend
MHC II	M5/114.15.2	eBioscience
B220	RA3-6B2	Biolegend
CD3	17A2	Biolegend
CD11c	HL3	BD Biosciences

Abbreviation: MHC II, major histocompatibility complex II.

## Supplementary Materials and Methods

### Mice

The following complement-deficient strains, backcrossed to BALB/c for 10 generations, were used: *C3*^*−/−*^ (Wessels et al., 1995), *C1qa*^*−/−*^ (Botto et al., 1998), *C5ar1*^*−/−*^ (Höpken et al., 1996), *C5ar2*^*−/−*^ (Gerard et al., 2005), and *C3ar1*^*−/−*^ (Humbles et al., 2000). C6^*−/−*^ mice were on a C57BL/6 background (Bhole and Stahl, 2004). Mice were age-matched and sex-matched for each experiment and used between 8 and 12 weeks of age. For 7,12-dimethylbenz[a]anthracene (DMBA)‒12-O-tetradecanoylphorbol13-acetate tumor experiments, all mice were initiated with DMBA at 8 weeks of age. The mice were selected from a large pool without any specific method of randomization. Wild-type BALB/c or C57BL/6 controls were purchased from Charles River Laboratories (Wilmington, MA). The mice were kept in specific pathogen-free conditions and handled in accordance with the institutional guidelines. All procedures were approved by the Imperial College Animal Welfare and Ethical Review Body committee, the United Kingdom Home Office, and conducted in accordance with the Animal Research: Reporting of In Vivo Experiments guidelines (Percie du Sert et al., 2020). Experiments involving cancer studies strictly adhered to the guidelines set out by the National Cancer Research Institute and in the document *Guidelines for the Welfare and Use of Animals in Cancer Research* (Workman et al., 2010).

### Chemical-induced carcinogenesis

Chemical carcinogenesis protocols were conducted as previously described (Strid et al., 2008). Briefly, the dorsal back skin of 7-week-old mice was shaved under general anesthesia using electric clippers, and mice were allowed to rest for 1 week. For DMBA-12-O-tetradecanoylphorbol13-acetate carcinogenesis, DMBA was applied topically to the shaved area at a dose of 600 nmol in 150 μl acetone. After 1 week, 12-O-tetradecanoylphorbol13-acetate was applied topically at a dose of 20 nmol twice weekly (BALB/c mice) or 15 nmol thrice weekly (C57BL/6 mice). For DMBA-only carcinogenesis, an initial dose of 200 nmol DMBA was applied in 100 μl acetone, followed by once-weekly doses of 100 nmol. Additional shaving was performed as required to prevent hair regrowth. Mice were monitored regularly, and cutaneous tumors were scored, counted, and measured with a caliper.

### Neutrophil depletion

To deplete blood neutrophils, anti-Ly6G antibody was injected intraperitoneally as previously described (Daley et al., 2008). Mice were injected with 200 μg of either rat IgG2a anti-Ly6G (Clone 1A8, Biolegend, San Diego, CA) or a chimeric anti-Ly6G composed of the 1A8 variable region with a mouse IgG2a fragment-crystallizable region to avoid eliciting a neutralizing response (absolute antibody). The antibody was injected at the time points indicated in the figure legends. Blood neutrophil depletion was validated by flow cytometry, with neutrophils defined independently of their Ly6G expression as SSC^high^, CD11b^pos^, Ly6C^int^, CD115^neg^.

### Histology and immunofluorescence of mouse skin

Ear skin was harvested from naïve or 12-O-tetradecanoylphorbol13-acetate–treated mice and fixed in 10% PBS-buffered formalin for 24 hours before transfer to 70% ethanol. Samples were embedded in paraffin, and 3 μM sections were stained with H&E. Images were acquired using a Leica DM4B microscope (Leica Microsystems, Wetzlar, Germany) with either a ×20/0.4 or a ×10/0.25 objective. Image analysis and processing were performed in ImageJ (Schindelin et al., 2012). To assess the dorsal epidermal thickness, samples were blinded, and enough fields were acquired to cover the entire length of the ear skin. For each field, 10 measurements were taken from the basement membrane to the stratum corneum at equal intervals. The mean of the total measurements was then calculated for each biological replicate.

Tissue (tumors or ear skin) was harvested and snap frozen in optimal-cutting temperature using isopentane cooled to −80 °C on dry ice. The 9 μM sections were cut using a Leica JUNG CM1800 cryostat (Leica Microsystem) and stored at –80 °C. For staining, the slides were equilibrated to room temperature before fixation in ice-cold acetone for 10 minutes. Sections were blocked in PBS with 5% BSA and 10% serum (dependent on the species of the secondary antibody being used) for 1 hour at room temperature. All antibody-staining steps were performed in PBS-5% BSA-0.1% Triton X-100 for 45 minutes at room temperature. The following primary antibodies were used: goat anti-C3 FITC (MP Biomedicals, Irvine, CA), goat anti-C3d biotin (R&D Systems, Minneapolis, MN), rat anti-CD49f (clone GoH3, Biolegend), rabbit anti-cytokeratin 10 (clone EP1607IHCY, Abcam, Cambridge, United Kingdom). Hoescht 33342 (NucBlue, ThermoFisher, Waltham, MA) was added to the final staining step according to the manufacturer’s instructions, and sections were mounted in ProLong Glass (ThermoFisher). Images were acquired on a Zeiss Axio Observer-inverted widefield microscope (Zeiss, Oberkochen, Germany) using a Colibri.2 LED illumination source, a ×20/0.8 plan-apochromat objective and a Hamamatsu Flash 4.0 camera. Images were processed to correct brightness and contrast in FIJI (Schindelin et al., 2012).

### Immunohistochemical and immunofluorescent staining of human skin

Immunohistochemistry was carried out on formalin-fixed, paraffin-embedded human tissue sections. Samples were collected according to the Helsinki Declaration, and the study was approved by the University of Palermo Ethical Review Board (approval number 09/2018). Tissue from five patients with cutaneous squamous cell carcinoma and two normal skin biopsies were analyzed. Briefly, sections 2.5 of 3 micron-thick were cut from paraffin blocks, dried, dewaxed, and rehydrated. The antigen-unmasking technique was performed using Novocastra Epitope Target Retrieval Solutions (Novocastra, Newcastle upon Tyne, United Kingdom), pH9 EDTA-based buffer in a thermostatic bath at 98 °C for 30 minutes. Subsequently, the sections were brought to room temperature and washed in PBS. After neutralization of the endogenous peroxidase with 3% hydrogen peroxide and fragment-crystallizable blocking by a specific protein block (Novocastra), tissue samples were incubated with a rabbit polyclonal anti-C3d antibody (1:100 pH9; Cell Marque, Code 403A-76). The immunostaining was revealed by a polymer-detection method (Novolink Polymer Detection Systems, Leica Biosystems, product number RE7280-K). The slides were counterstained with Harris hematoxylin (Novocastra).

For the immunofluorescence, the sections were incubated with the following primary antibodies: rabbit polyclonal anti-C3d (1:100 pH9; Cell Marque, Code 403A-76) and mouse monoclonal anti-CD163 (clone 10D6; 1:100 pH9; Leica Biosystem). Bound antibodies were revealed by the following fluorochrome-conjugated secondary antibodies: Alexa Fluor 568‒conjugated goat anti-rabbit IgG (H+L) (1:300 dilution) and Alexa Fluor 488‒conjugated goat anti-mouse IgG (H+L) (1:250 dilution), both from Invitrogen Molecular Probes (Carlsbad, CA). Nuclei were counterstained with DAPI.

All the sections were analyzed under a Zeiss AXIOScope.A1 optical microscope (Zeiss), and microphotographs were collected using a Zeiss Axiocam 503 Color digital camera using the Zen 2.0 imaging software (Zeiss).

### qRT-PCR and primer sequences

Ear skin was harvested and split into the dorsal and ventral side using forceps. To separate dermis and epidermis, the dorsal side was floated dermis down on 0.5 M ammonium thiocyanate for 40 minutes at 37 °C. Whole-epidermal sheets were carefully lifted from the dermis, and both were washed in PBS. RNA was extracted with an RNeasy Mini Kit (Qiagen, Hilden, Germany) according to the manufacturer’s instructions. RNA was dissolved in RNase-free water, and concentration and purity were determined using a NanoDrop 2000 (ThermoFisher). cDNA was synthesized from RNA using an iScript cDNA synthesis kit (Bio-Rad, Hercules, CA) according to the manufacturer’s instructions. All primers were single-stranded DNA oligonucleotides (Sigma-Aldrich, St. Louis, MI) designed using ApE software (Wayne Davis, University of Utah, Salt Lake City, UT). Real-time PCR products were detected with SYBR Green (ThermoFisher) measured continuously with a ViiA 7 Real-Time PCR system (Applied Biosystems, Foster City, CA). Target gene expression was quantified using a six-point standard curve derived from pooled cDNA and normalized to the mean of two housekeeping genes encoding GAPD and 18S ribosomal RNA. Primer sequences are listed below.

1. *18S* (F): 5′ GTAACCCGTTGAACCCCATT 3′;
2. *18S* (R): 5′ CCATCCAATCGGTAGTAGCG 3′;
3. *GAPD* (F): 5′ TGTGTCCGTCGTGGATCTGA 3′;
4. *GAPD* (R): 5′ TTGCTGTTGAAGTCGCAGGAG 3′;
5. *C3* (F): 5′ CAAACAGATGTCCCTGACCA 3′;
6. *C3* (R): 5′ TGCTTTTGAGTTTGGCATGA 3′.

### Nanostring analysis

For gene expression in squamous cell carcinoma tumors, formalin-fixed, paraffin-embedded skin samples were obtained from 56 patients at the University of Dundee (United Kingdom), Tayside National Health Service Trust, and Greater Glasgow and Clyde National Health Service Trust. The study was conducted according to the Declaration of Helsinki Principles, and all patients donating samples to this study provided written informed consent in accordance with ethical approval from the East of Scotland Research Ethics Service REC 1. Squamous cell carcinoma tumors were graded by an experienced dermatopathologist as low risk or high risk on the basis of the Scottish Intercollegiate Guidelines Network for cutaneous squamous cell carcinoma. In addition, perilesional skin (histopathologically normal-looking skin more than 4 mm away from the tumor edge) and abdominal non–UV-exposed skin were also analyzed. Total RNA was extracted using a Qiagen RNeasy extraction kit and was directly hybridized using the NanoString PanCancer and PanCancer Immune expression panel and analyzed on an nCounter (NanoString, Seattle, WA).

### Tissue digestion and flow cytometry

Single-cell suspensions from ear skin were obtained as follows: (i) ears were split dorsal-ventral using forceps to analyze only the dorsal (treated) side; (ii) skin was cut into ∼1 mm^2^ pieces using a scalpel blade and incubated for 2 hours in digestion buffer containing 25 μg/ml Liberase (Roche, Basel, Switzerland), 250 μg/ml DNAseI (Roche), and 1 × DNase buffer (1.21 Tris base, 0.5 g magnesium chloride, and 0.073 g Calcium chloride) at 37 °C; (iii) pieces were transferred into C-tubes (Miltenyi Biotech, Bergisch Gladbach, Germany) containing RPMI-1640 medium (Thermo Fisher) supplemented with 10% heat-inactivated fetal calf serum and physically disrupted using a GentleMACS dissociator (Miltenyi Biotech); (iv) cell suspensions were then filtered through 70 μM cell strainers (BD Bioscience) and counted using a CASY cell counter (Roche). Cells were incubated with a saturating concentration of 2.4G2 mAb (anti-CD16/32) to block nonspecific fragment-crystallizable receptor binding and stained in PBS solution containing 1% BSA, 2 mM EDTA, and 0.09% sodium azide (FACS buffer). For skin staining, cells were initially incubated with a live/dead discrimination dye as per manufacturer’s instructions (LIVE/DEAD Fixable Dead Cell Stain, Thermo Fisher). For blood staining, tail vein blood samples were added to an equal volume of 100 mM EDTA, washed, and resuspended in FACS buffer. Cell-surface staining was then performed using the fluorochrome-conjugated antibodies listed in Supplementary Table S1. After antibody staining, cells were washed, and red blood cells were removed by hypotonic lysis using BD FACS Lysing Solution (BD Biosciences). Samples were analyzed on a Fortessa X20 flow cytometer (BD Biosciences) using FlowJo X for Mac (TreeStar, Woodburn, OR).

### Statistical analysis

Experimental groups were compared using two-tailed Student’s *t*-test for unpaired data. For experiments with more than two groups, unpaired data were analyzed using a one-way ANOVA with Tukey’s multiple comparison. Tumor burden between groups was assessed by linear regression, whereas tumor onset was analyzed using a Mantel‒Cox test. Asterisks denote significance as follows: ∗*P* < 0.05; ∗∗*P* < 0.01; ∗∗∗*P* < 0.001; and ∗∗∗∗*P* < 0.0001. Statistical tests were performed using GraphPad Prism 7.0 for Mac (GraphPad Software, La Jolla, California).
